# Some Established Principles in Prosthetic Dentistry

**Published:** 1897-05

**Authors:** L. P. Haskell

**Affiliations:** Chicago.


ARTICLE VI.

### SOME ESTABLISHED PRINCIPLES IN PROSTHETIC DENTISTRY.

BY L. P. HASKELL, CHICAGO.

### PLASTER IMPRESSIONS.

The more difficult the case of which to obtain an impression the greater the necessity for using plaster.

### AN INVARIABLE RULE.

Where the cuspid teeth have been extracted a year there is necessity and room for wearing the plate higher, and the artificial gum fuller, than elsewhere, to restore the contour of lip.

### VACUUM CAVITIES.

In full cases, upper permanent, there is no need of air-chambers or vacuum cavities, except in rare instances. This is asserted after thirty years non-use of them, having had every conceivable shape and condition of jaws to deal with, and in the heaviest continuous gum sets. Often they interfere with suction. In this connection it should be considered that the center of the palate is hard and the only portion of the upper jaw that never changes from absorption or pressure. As the alveolar ridge is constantly liable to change (especially under rubber) gradually the plate rests and rocks on this hard center. In metal plates, a thin "relief" of wax, the edges flush with the model, is needed in all cases, except in less than one per cent. where it is soft, and no change whatever is needed. This has been my invariable practice for thirty years, and the results are eminently satisfactory.

### DIE METAL.

Nothing has so simplified the fitting of metal plates as the use of Babbitt metal properly made. It would seem as though the exclusive use of this metal for forty-five years, after having used everything else ever used, would establish this fact. One writer in a recent work objects to it on account of expense. Five pounds costing $2.50 would last an ordinary practice a year. Babbit metal is sufficiently hard and tough for all cases. If it cracks it shows a lack of tin, the addition of a little will remedy it. The proper formula is copper one part, antimony two parts, tin eight parts, to be melted in the order named, otherwise the tin will oxidize in mixing.

As pure lead can not be poured on Babbit metal the melting temperature of the lead is reduced by adding one-sixth tin, and as lead is too soft for the counter-die, the tin hardens it. The die and counter-die should never be of the same metal, as neither will yield, and the softer metal (the plate) will be torn, or made very thin at prominent points, and their use is unnecessary. A second die of Babbit metal is seldom needed, and a second counter never.

### OILED SAND.

Its advantages are two-fold, viz.: When once prepared it can be used many times without re-oiling, thus saving time and annoyance. When moistened with water, if too wet or packed so hard the steam cannot escape, blow-holes are liable in the die. This never occurs with oiled sand. It should never be used with zinc, as that is poured so hot it burns the sand too much. To the objections sometimes made to the "odor and soiling the hands," it may be suggested that these are discounted by the opening and handling of a vulcanite flask.

### Moulding Flasks.

The Bailey moulding flasks are unfit for the purpose, too small and too flaring. More room is needed to pack conveniently. Have them made of heavy sheet iron, three inches deep and four inches diameter.

### The Use of the Blow-pipe.

It is strange, but true, that the blow-pipes furnished the dental profession from the earliest days to the present are simply jewelers' blow-pipes, and unfit for dentists' use.

The jeweler uses low-grade solders, and has no investment to contend with. The pipe is so small it has to be taken inside the lips, tiring the muscles.

The dentist with his high-grade solders and heavy investments needs a large blow-pipe, the mouth of which is pressed against the lips, so that the blowing is made easy; then with the larger orifice at the heat end a larger flame can be secured. Years ago in Boston I had a mandrel made on which I made such a blow-pipe for the profession. In the later years the dental goods dealers have at my request made such a blow-pipe which they have named the "Haskell."

I have found in teaching students they could learn easily the use of the mouth blow-pipe, and succeed better as beginners in metal work than with the automatic.

### Vulcanized Rubber.

The most serious objections to the use of rubber for full upper dentures is its non-conductibility; the retention of undue heat, causing constant change in the process, so that in thousands of cases there is no ridge left, or only a ridge of thickened membrane. Dr. George Watt's theory was that the retention of undue heat did not cause additional absorption, but what was practically the same in results, prevented a replacement of lost tissue.

### REPAIRING RUBBER PLATES.

The old method of repairing by means of dove-tails, holes and solutions has long since been discarded by progressive workmen, and in their stead a simpler method pursued. If a broken plate, fasten the parts together with wax, and fill the plate with plaster; if a missing tooth, wax one in place and flask only one-half of a flask. In both cases remove wax and clean the plate; in the broken plate cut away a portion of fracture, thinning the edges for a distance of one-quarter inch or more, leaving at extreme margin a depression of 1-32 inch. With a hot spatula press fresh rubber on to the surface and fill to the desired fullness, flask and vulcanize, and the union will be found perfect. In the replacing of the tooth use the hot spatula and finish flasking.

### ALUMINUM FOR PLATES.

This makes an excellent cheap plate. To the objections sometimes made that it is acted on by a caustic solution of soda and is therefore unfit for use in the mouth, it might be said that the oral cavity does not contain a "caustic solution of soda" nor anything else that will deteriorate the metal. I have never seen the first evidence of such action in any plate after several years' use. As formerly reduced the metal contained little specs of iron which rusted, and holes resulted. The present metal is entirely free from any impurities. By the use of the "loop-punch" the rubber is firmly held to the plate.

### ARTICULATING TEETH.

There are more failures from faulty articulation than any other cause.

The six anterior teeth should never come in contact resulting as it does in crowding the upper plate forward and down at the back.

Pressure should be exact on both sides on bicuspids and first molars.

If a lower, second or third molar is an inclined plane, do not allow the upper molar to come into close contact.

### Continuous Gum Work.

This work, after nearly fifty years' use, has no peer as a full denture. Nothing ever made approaches it as the strongest, most desirable, most natural in appearance, most healthy to the oral tissues and the most cleanly, when properly made.

Weight cuts no figure, except in rare cases.—*Ohio Dental Journal.*

